# Spontaneous Tumor Lysis Syndrome in T-Cell Leukemia

**DOI:** 10.7759/cureus.11002

**Published:** 2020-10-17

**Authors:** Beenish Faheem, Sudarsan Kollimuttathuillam, Hamdallah Ashkar, Michael Maroules

**Affiliations:** 1 Internal Medicine, St. Joseph's University Medical Center, Paterson, USA; 2 Hematology/Oncology, St. Joseph's University Medical Center, Paterson, USA

**Keywords:** acute spontaneous tumor lysis syndrome, t-cell leukemia, lymphoma, chemoradiation therapy, hematologic malignancy, tumor lysis syndrome, precipitated without previous chemotherapy, acute lymphoblastic leukemia, spontaneous tumor lysis without precipitating factor, hyperkalemia

## Abstract

Tumor lysis syndrome (TLS) is a constellation of metabolic derangements that occur as a consequence of rapid cell turnover in malignancy and the release of intracellular solutes - potassium, phosphate, and nucleic acid metabolites. TLS classically occurs following chemotherapy, with severe renal failure. However, the entity referred to as spontaneous TLS occurs without a precipitating factor of chemotherapy, radiotherapy, steroid therapy, or immunotherapy and can develop in both hematologic and solid malignancies. Here, we report a rare case of a patient who presented with nonspecific symptoms, hyperphosphatemia, hyperuricemia, but hypercalcemia, resultant acute renal failure, with a large mediastinal mass and a pericardial effusion, who was later found to have spontaneous TLS. The workup led to the diagnosis of T-cell leukemia.

Spontaneous TLS is often the first manifestation of occult or undetected malignancy, making this oncologic emergency a challenge to manage. The early diagnosis and prompt treatment of spontaneous TLS can reduce morbidity and mortality for patients with an otherwise curable disease.

## Introduction

Acute tumor lysis syndrome (TLS) is a true oncological emergency and may likely be the most significant cause of acute renal failure in cancer patients [1]. TLS is a constellation of metabolic derangements that occur as a consequence of rapid cell turn over in malignancy and the release of intracellular solutes - potassium, phosphate, and nucleic acid metabolites [1]. TLS classically occurs following chemotherapy, with severe renal failure. However, the entity referred to as spontaneous TLS occurs without a precipitating factor of chemotherapy, radiotherapy, steroid therapy, or immunotherapy and can develop in both hematologic and solid malignancies [1-2]. Here, we report a rare case of a patient who presented with nonspecific symptoms, hyperphosphatemia, hyperuricemia, but hypercalcemia with a large anterior mediastinal mass leading to a pericardial effusion, who was later found to have spontaneous TLS. The workup led to the diagnosis of T-cell acute lymphoblastic leukemia.

Spontaneous TLS is often the first manifestation of occult or undetected malignancy, making this oncologic emergency a challenge to manage [2]. Therefore, when presented with acute kidney impairment and dramatic metabolic derangements, clinicians should be prompted to consider spontaneous TLS as a diagnosis, even if a malignancy has not yet been diagnosed in the patient. The early diagnosis and prompt treatment of spontaneous TLS can reduce morbidity and mortality for patients with an otherwise curable disease.

## Case presentation

A 50-year-old Italian-American man with a past medical history of hypothyroidism and noncompliance with thyroxine was found to have acute renal insufficiency (baseline creatinine 0.85-1.0) with a white blood cell count (WBC) of 13.4 x 10^9^/L (normal range: 4.5-11.0 x 10^9^/L) and a creatinine of 3.47 mg/dL (normal range: 0.6-1.3 mg/dL) on admission. At the time of admission, the patient reported a one-week history of exertional dyspnea, a three-week history of increasing abdominal girth with night sweats, and subjective fevers. He reported a family history significant for pancreatic cancer in his grandfather and uterine cancer in his grandmother. The patient stated that one year prior to admission, his last visit with his primary care physician revealed normal labs.

Physical examination demonstrated a patient in no acute distress with blood pressure (BP) of 145/78 mmHg, a heart rate (HR) of 90 beats/min, and respiratory rate (RR) of 18 breaths/min. Marked hepatosplenomegaly was noted without palpable cervical, supraclavicular, or inguinal lymphadenopathy. The basic metabolic panel revealed (corrected calcium) hypercalcemia of 12.9 mg/dL (normal range: 8.3-10.6mg/dL), acute kidney injury blood urea nitrogen (BUN) of 73 mg/dL (normal range: 7-23 mg/dL), creatinine 3.47 mg/dL, hyperphosphatemia of 6.1 mg/dL (normal range: 2.5-5.0 mg/dL), hyperuricemia of 18.7 mg/dL (normal range: 3.4-7.0 mg/dL), elevated lactate dehydrogenase of 2498 units/L (normal range: 140-271 units/L), elevated transaminases with aspartate aminotransferase of 192 units/L (normal range: 13-39 units/L), and alanine aminotransferase of 124 units/L (normal range: 7-52 units/L). His complete blood count revealed tri-lineage hematopoietic dysfunction with thrombocytopenia with platelets of 50,000/mcL (normal range: 140,000-440,000/mcL), lymphopenia, and monocytosis with peripheral blood smear that revealed promyelocytes, myelocytes, metamyelocytes, atypical lymphocytes, and basophilia.

**Figure 1:**
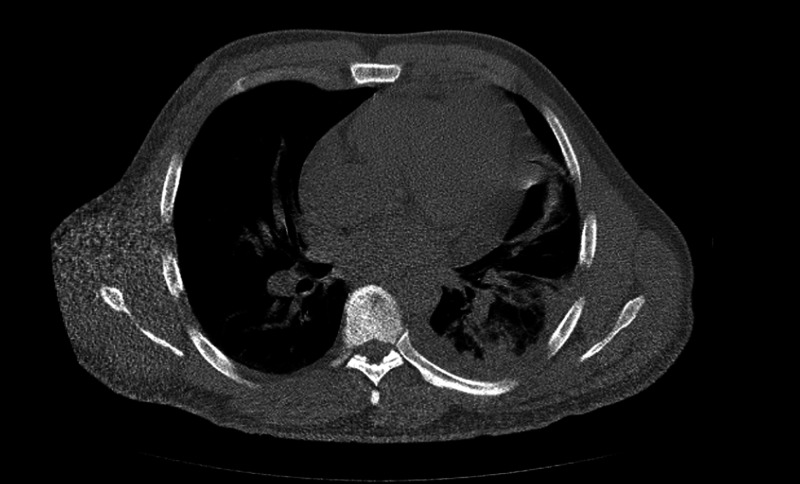
Anterior mediastinal heterogenous mass measuring 12 cm x 7 cm x 8 cm with a mass effect on the heart and great vessels

**Figure 2:**
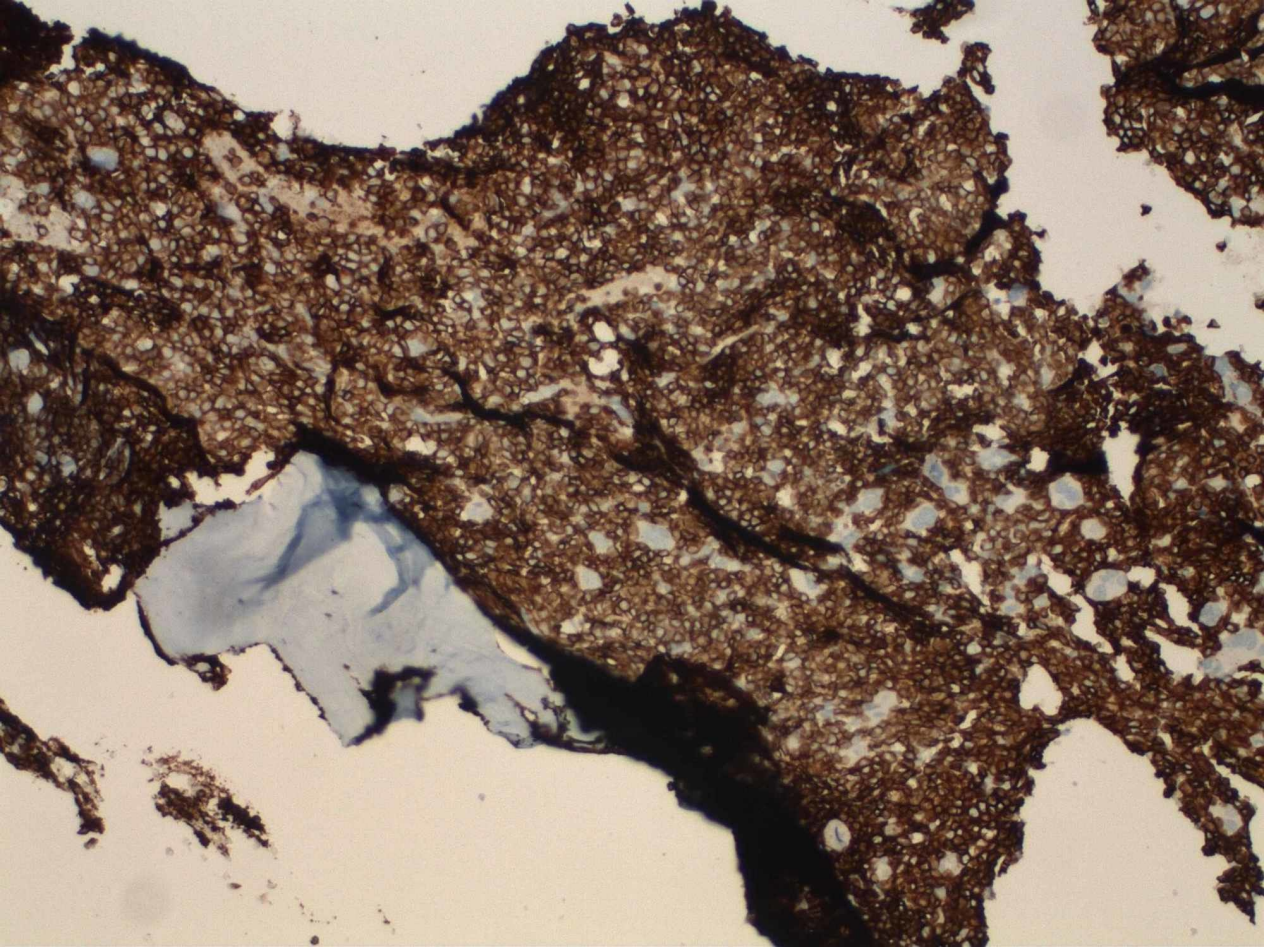
Acute lymphoblastic leukemia bone marrow with immunohistochemical stains: CD3, TdT, CD1a, CD99, and CD5 CD: cluster of differentiation; TdT: terminal deoxynucleotidyl transferase

**Figure 3:**
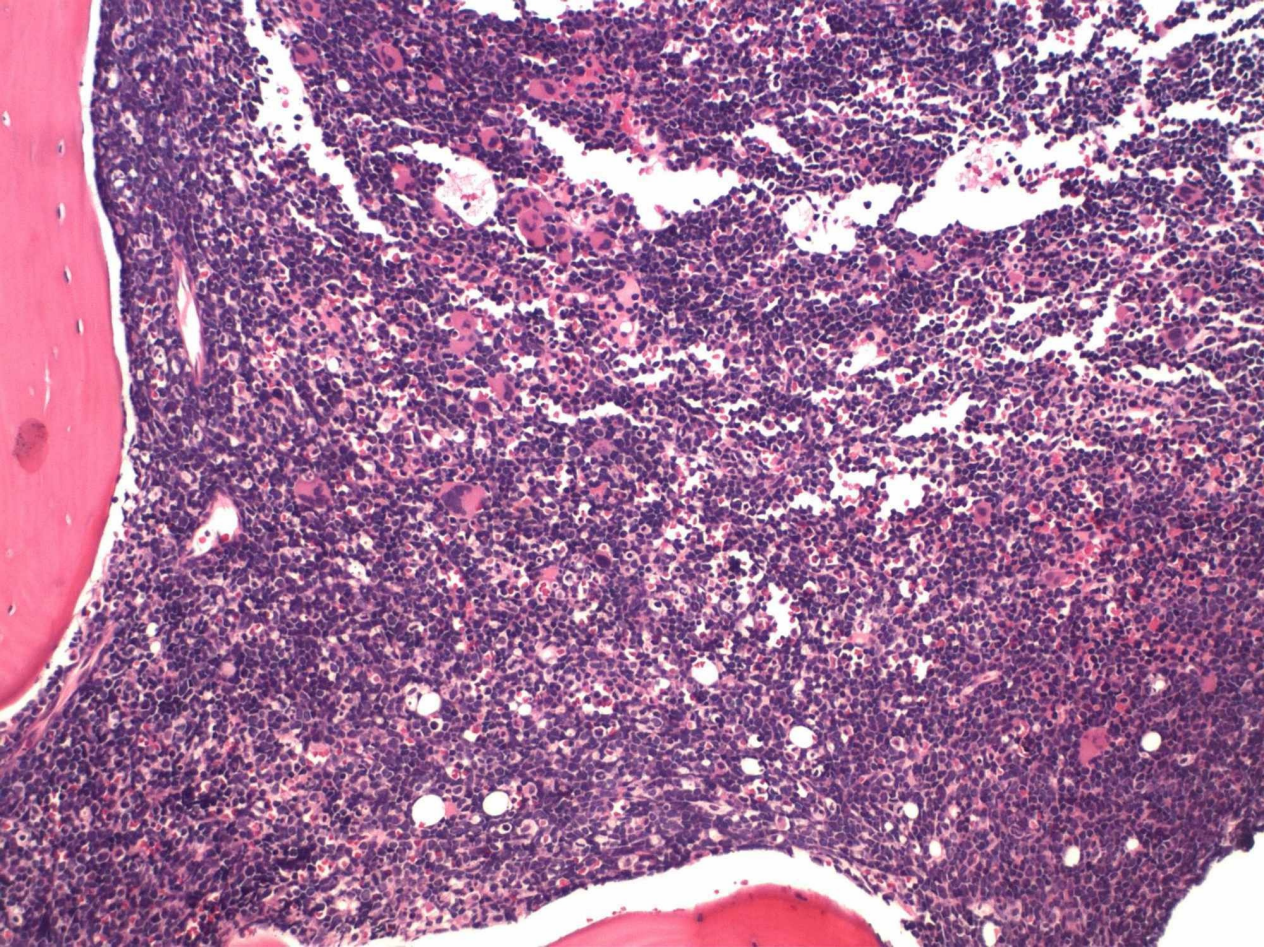
Acute lymphoblastic leukemia bone marrow H&E stain showing diffuse proliferation of lymphoid cells of medium to large-sized immature cells, replacing approximately 90% marrow cellularity, with leukemic blasts and high nuclear to cytoplasmic ratio H&E: hematoxylin and eosin

A computed tomography (CT) scan revealed a heterogeneous mass measuring 12 x 7 x 8 cm with a mass effect on the heart (Figure 1) and great vessels with marked hepatosplenomegaly. The patient received aggressive hydration along with rasburicase and allopurinol. The patient’s soluble interleukin-2 levels were in 8621. Leukemia was suspected. The patient's kidney function worsened on day four of hospitalization. With gradually worsening hyperkalemia, hyperphosphatemia, and hyperuricemia, spontaneous tumor lysis syndrome was suspected and emergent hemodialysis was initiated. The patient was empirically started on decadron. A bone marrow biopsy was conducted. Histopathological examination of the lymph node revealed diffuse proliferation of lymphoid cells of medium to large-sized immature cells, replacing ~90% marrow cellularity. The biopsy showed leukemic blasts with a high nuclear to cytoplasmic ratio, dispersed chromatin, and slightly irregular nuclear contour. On immunostains, leukemic cells are positive for cluster of differentiation 3 (CD3), terminal deoxynucleotidyl transferase (TdT), CD1a, CD99, and CD5 (variable intensity). The blasts are negative for CD20, CD34, CD117, CD10, and CD30. See Figures 2-3 for the results of immunostaining.

The flow cytometry analysis revealed an abnormal immature population of T-cells with both CD4 and CD8 expression. Based on these histopathological findings, in the setting of mediastinal mass and hepatosplenomegaly, a diagnosis of T-cell acute lymphoblastic leukemia was made. By day six, the patient’s clinical condition further deteriorated, with worsening thrombocytopenia leading to epistaxis and hemoptysis requiring desmopressin and repeated hemodialysis for a potassium level of 8.7 mg/dL (normal range: 3.5-5.0 mg/dL). Despite aggressive measures on day seven, the patient expired due to a cardiac arrest.

## Discussion

TLS is typically associated with high-grade, poorly differentiated lymphomas and acute lymphoid leukemia [1]. It is rarely reported in association with acute myeloid leukemia (AML) and other hematologic malignancies. Spontaneous TLS occurs prior to the administration of cytotoxic therapies, such as chemotherapy, immunotherapy, and radiotherapy, manifesting with severe electrolyte derangements and renal failure as a consequence [1-2].

TLS is both a renal and an oncologic emergency. TLS is a consequence of massive cell turnover of tumor cells as a result of chemotherapy releasing their intracellular contents into the serum, producing the typical laboratory abnormalities that include hypocalcemia, hyperuricemia, hyperkalemia, and hyperphosphatemia [1-2].

The massive destruction of neoplastic cells serves as an intrinsic cause of acute renal failure. It is most commonly observed as a complication of treating hematologic malignancies with chemotherapy, for example, Burkitt’s lymphoma, acute lymphoid leukemia, and chronic lymphocytic leukemias as well as non-hematologic solid malignancies [3]. A high WBC count, elevated serum lactate dehydrogenase (LDH), acute renal failure, and hyperuricemia are risk factors for developing TLS. However, the entity - spontaneous TLS - is rarely observed among hematologic malignancies without the precipitating factors of chemotherapy, radiation, or immunotherapy [2]. Spontaneous tumor lysis syndrome is described as tumor lysis occurring in the absence of definitive chemotherapy [2]. It was reported in patients with Burkitt’s lymphoma/leukemia, diffuse large B-cell lymphoma, anaplastic large T-cell lymphoma, acute myeloid leukemia, adenocarcinoma of the lung, breast cancer, gastric cancer, and myelofibrosis [3-5]. Here, we present a rare case of spontaneous TLS associated with T-cell leukemia of acute lymphoblastic leukemia (ALL). To our knowledge, only a few cases of severe spontaneous acute TLS with ALL have been reported in the literature, with only one other case of acute spontaneous TLS and a large mediastinal mass with pericardial effusion together [4]. Typically, pericardial effusion and cardiac tamponade may develop as complications of leukemia secondary to radiation therapy or chemotherapy or they may develop due to the involvement of the pericardium as a manifestation of perivascular infiltration [4]. Our patient had pericardial effusion without any therapy or infection. Transthoracic echocardiography detected a large mediastinal mass surrounding the heart. It is likely that the pericardial effusion could have developed as a result of the extension of the lymphoma from the large anterior mediastinal mass [4].

The exact mechanism of how spontaneous TLS occurs remains unclear [2,4]. It has been suggested that rapid tumor necrosis releasing intracellular metabolites may be a component in the development of spontaneous TLS [6-7]. TLS is the main cause of acute renal injury among cancer patients [3]. Renal failure is precipitated by uric acid deposition in the kidney tubules due to hyperuricemia caused by increased cell turnover. Spontaneous TLS has been noted to be the first manifestation of an occult malignancy [2]. There have been cases reported where hyperphosphatemia and hypocalcemia appear less frequently with spontaneous TLS. As such, in our patient’s case, there was, in fact, hypercalcemia (12.5 mg/dl), therefore, rendering the diagnosis of spontaneous TLS more challenging to make, as such underestimating the severity of the condition. Consequently, it can be postulated whether the current diagnostic criterion, widely accepted, is appropriate to identify and recognize this syndrome [2]. Several reports have demonstrated that some patients, similar to our patient, with spontaneous TLS, do not meet all diagnostic criteria in place. Therefore, there are studies that propose acute renal failure, elevated LDH, with hyperuricemia, as the only criteria perhaps required in the diagnosis of this syndrome, rather than fulfilling the electrolyte abnormalities [2]. It has been suggested that the diagnosis of spontaneous TLS should be high on the differential if undiagnosed malignancy is suspected given a patient’s clinical presentation along with laboratory findings showing increased cell turnover with end-organ failure [2]. Although this approach may be simplistic, it could facilitate earlier recognition and diagnosis of the syndrome, leading to decreased morbidity and mortality associated with undiagnosed spontaneous TLS. This is of great importance for all clinicians, as prompt recognition of TLS and subsequent management can be lifesaving.

## Conclusions

TLS is a rare condition that is seen in patients with underlying hematologic or solid tumors undergoing rapid cell turnover. As spontaneous TLS can be the first presenting feature of an occult malignancy, it is incumbent upon all physicians to be familiar with and maintain a high index of suspicion even when electrolyte derangements do not fulfill the typical criteria. Early diagnosis and prompt treatment can save lives in patients with an otherwise curable cancer.
